# Supplementary material for: Cocrystallization of Progesterone with Nitrogen Heterocyclic Compounds: Synthesis, Characterization, Calculation and Property Evaluation
Source: Molecules. 2023 May 22;28(10):4242. doi: 10.3390/molecules28104242 (PMC10224249; doi:10.3390/molecules28104242)
Supplement: Supplementary file 1 [file molecules-28-04242-s001.zip › Supplementary Materials.pdf]

Supporting Information

Cocrystallization of Progesterone with nitrogen heterocyclic compounds:Synthesis, characterization, calculation and property evaluation

Juan Xu[1#], Wei Gao[2#], Qi Zhang[3]*, Lifeng Ning [1]*

[1] NHC Key Laboratory of Reproductive Health Engineering Technology Research, National Research Institute for Family Planning, Beijing 100081, China.

[2] School of Pharmacy, Guangdong Pharmaceutical University, Guangzhou 510006, China.

[3] School of Chemistry and Chemical Engineering, Beijing Institute of Technology, Beijing 100081, China.

[#]J. Xu and W. Gao contributed equally to this article.

Content

Figure S1. Scanning electron microscope (SEM) of PRO and 6 cocrystals (10μm)

Figure S2. Fourier transform infrared spectroscopy (FT-IR) of PRO and 6 cocrystals

Figure S3. ^{1}H nuclear magnetic resonance spectra (^{1}H-NMR) of PRO and 6 cocrystals

Figure S4. Thermal analysis of PRO and 6 cocrystals

Figure S5. The stability experiment of PRO and 6 cocrystals under different conditions

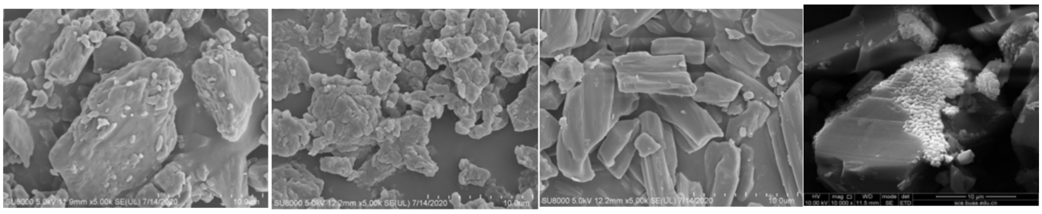

PRO PRO-DMP PRO-INA PRO-AP

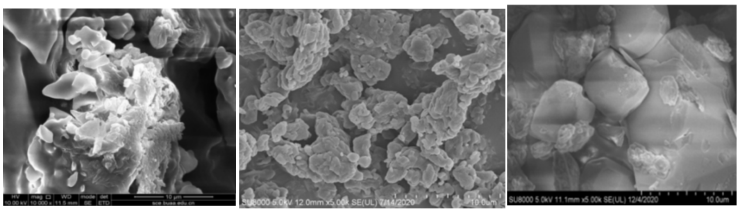

PRO-APZ PRO-PA PRO-PZA

Figure S1. Scanning electron microscope (SEM) of PRO and 6 cocrystals (10μm)

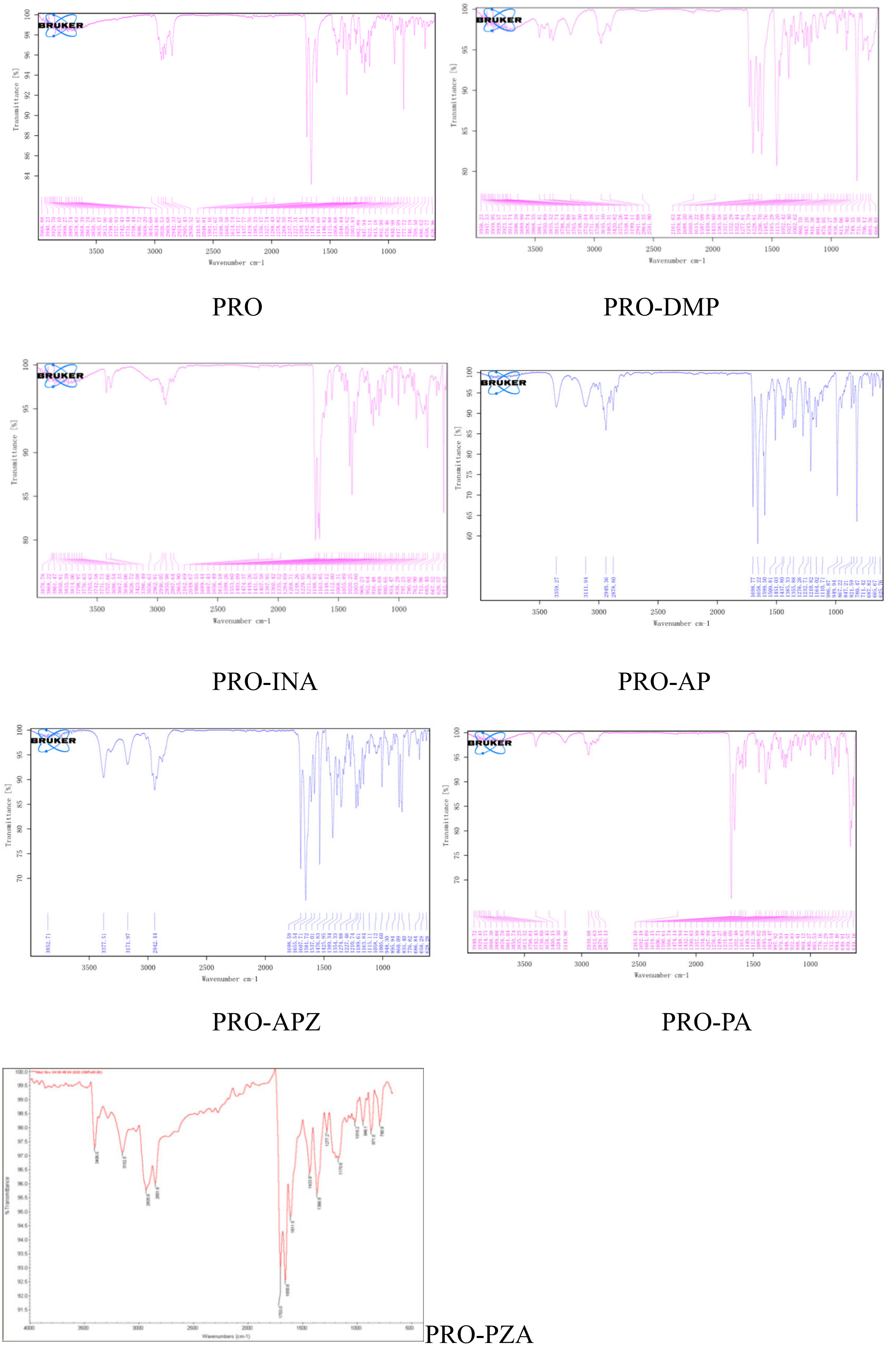

Figure S2. Fourier transform infrared spectroscopy (FT-IR) of PRO and 6 cocrystals

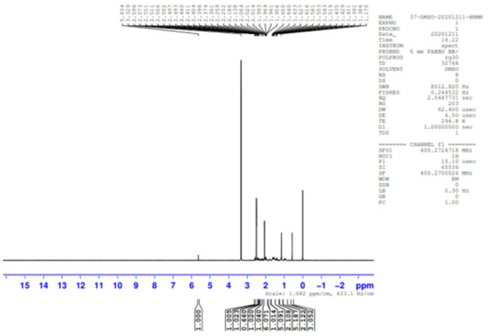

PRO

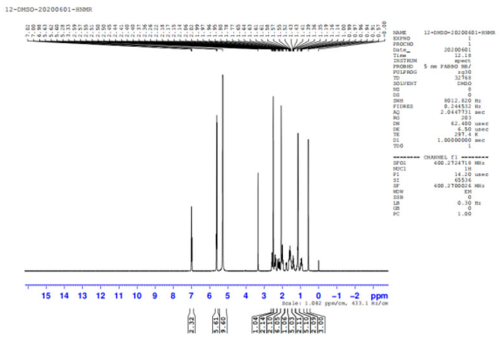

PRO-DMP

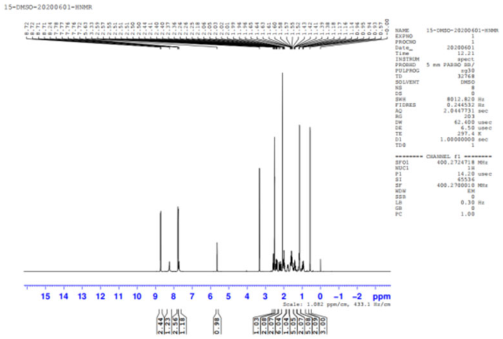

PRO-INA

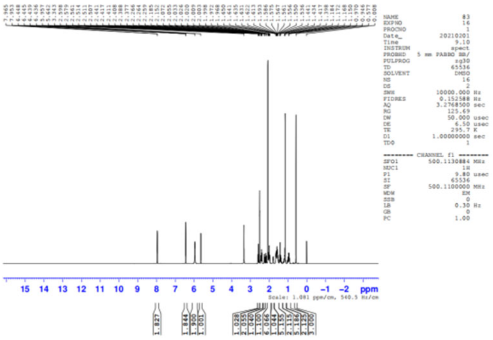

PRO-AP

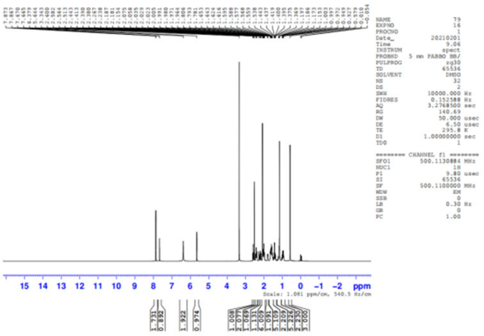

PRO-APZ

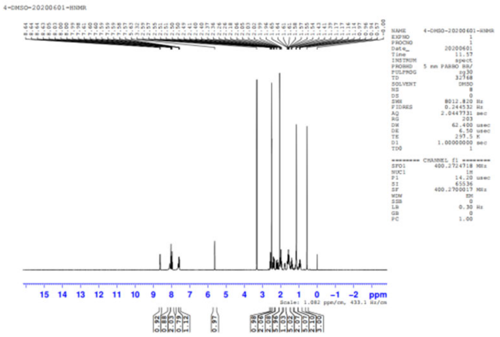

PRO-PA

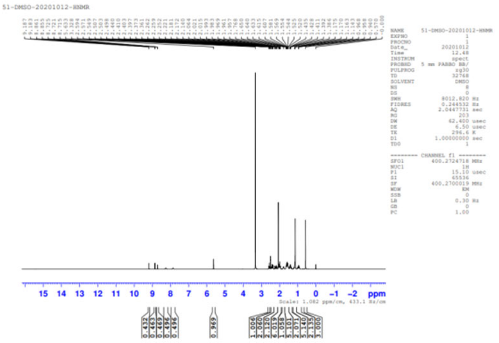

PRO-PZA

Figure S3. ^{1}H nuclear magnetic resonance spectra (^{1}H-NMR) of PRO and 6 cocrystals

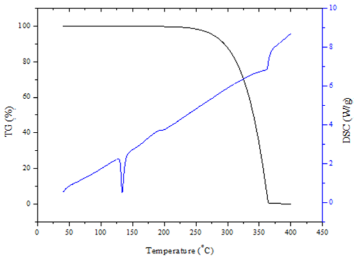

PRO

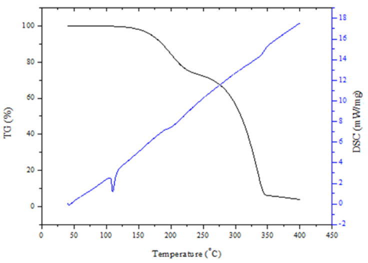

PRO-DMP

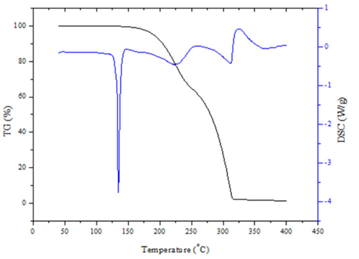

PRO-INA

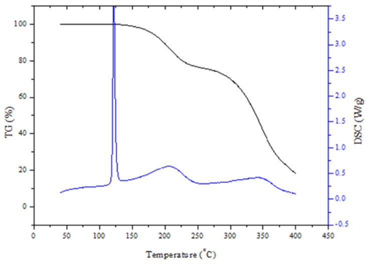

PRO-AP

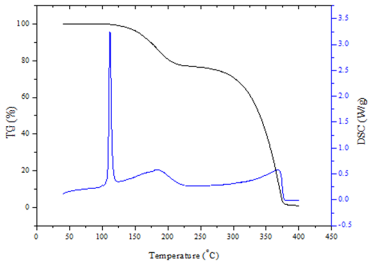

PRO-APZ

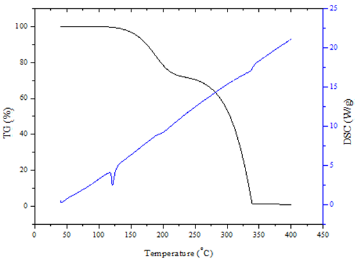

PRO-PA

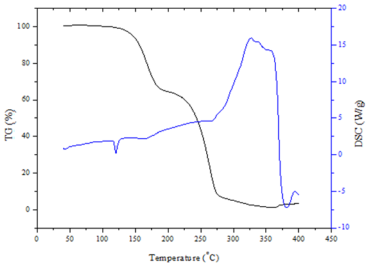

PRO-PZA

Figure S4. Thermal analysis of PRO and 6 cocrystals

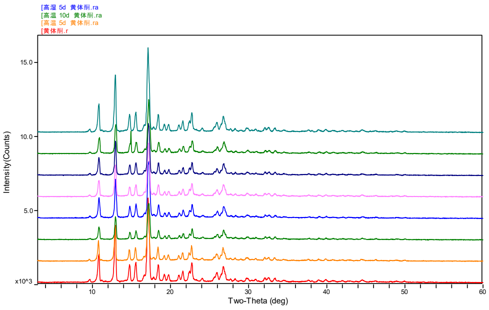

PRO

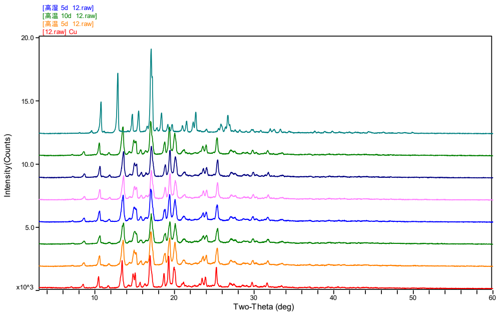

PRO-DMP

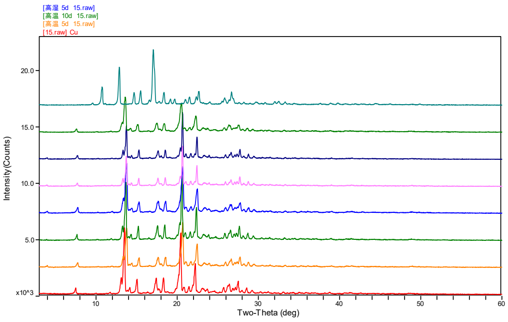

PRO-INA

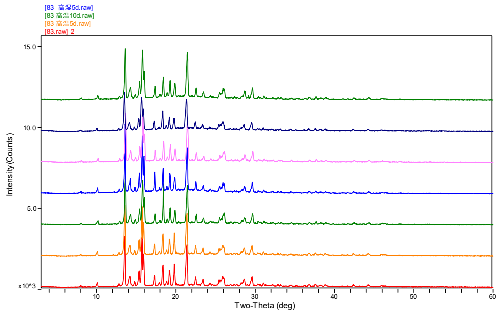

PRO-AP

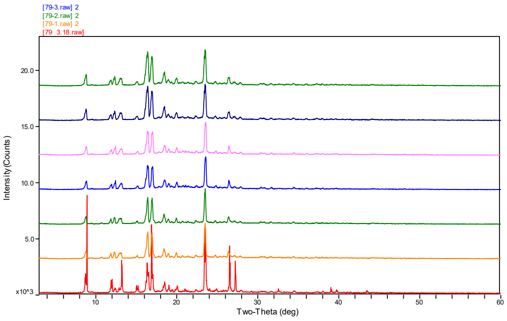

PRO-APZ

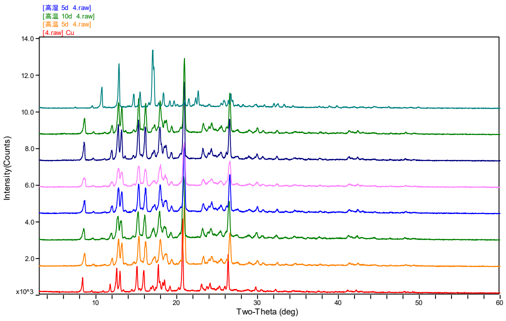

PRO-PA

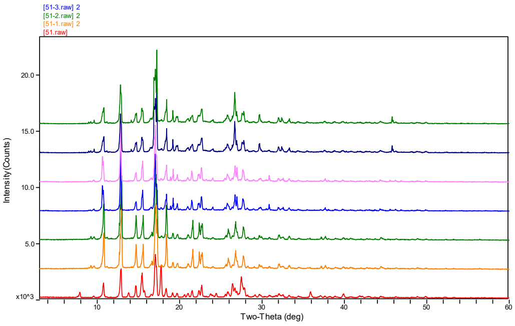

PRO-PZA

Figure S5. The stability experiment of PRO and 6 cocrystals under different conditions
